# Functional Interplay between P5 and PDI/ERp72 to Drive Protein Folding

**DOI:** 10.3390/biology10111112

**Published:** 2021-10-28

**Authors:** Motonori Matsusaki, Rina Okada, Yuya Tanikawa, Shingo Kanemura, Dai Ito, Yuxi Lin, Mai Watabe, Hiroshi Yamaguchi, Tomohide Saio, Young-Ho Lee, Kenji Inaba, Masaki Okumura

**Affiliations:** 1 Frontier Research Institute for Interdisciplinary Sciences, Tohoku University, 6-3, Aramakiaza Aoba, Aoba-ku, Sendai 980-8578, Japan; matsusaki@tokushima-u.ac.jp (M.M.); shingo.kanemura@kwansei.ac.jp (S.K.); mai.watabe.e4@tohoku.ac.jp (M.W.); 2 Institute of Advanced Medical Sciences, Tokushima University, 3-18-15, Kuramoto-cho, Tokushima 770-8503, Japan; saio@tokushima-u.ac.jp; 3 School of Science and Technology, Kwansei Gakuin University, 2-1, Gakuen, Sanda 669-1337, Japan; fic13812@kwansei.ac.jp (R.O.); fnc11916@kwansei.ac.jp (Y.T.); hiroshi@kwansei.ac.jp (H.Y.); 4 Department of Brain and Cognitive Science, Daegu Gyeongbuk Institute of Science and Technology, 333, Techno Jungang Daero, Daegu 42988, Korea; daiito2525@dgist.ac.kr; 5 Research Center for Bioconvergence Analysis, Korea Basic Science Institute, 162, Yeongudanji-ro, Ochang-eup, Cheongwon-gu, Cheongju-si 28119, Korea; linyuxi@kbsi.re.kr (Y.L.); mr0505@kbsi.re.kr (Y.-H.L.); 6 Bio-Analytical Science, University of Science and Technology, 217, Gajeong-ro, Yuseong-gu, Daejeon 34113, Korea; 7 Graduate School of Analytical Science and Technology, Chungnam National University, 99, Daehak-ro, Yuseong-gu, Daejeon 34134, Korea; 8 Research Headquarters, Korea Brain Research Institute, 61, Cheomdan-ro, Dong-gu, Daegu 41068, Korea; 9 Institute of Multidisciplinary Research for Advanced Materials, Tohoku University, 2-1-1 Katahira, Aoba-ku, Sendai 980-8577, Japan; kenji.inaba.a1@tohoku.ac.jp

**Keywords:** protein disulfide isomerase family, disulfide bond, endoplasmic reticulum, oxidative folding, molecular chaperone, protein-protein interaction

## Abstract

**Simple Summary:**

The physiological functions of proteins are destined by their unique three-dimensional structures. Almost all biological kingdoms share conserved disulfide-catalysts and chaperone networks that assist in correct protein folding and prevent aggregation. Disruption of these networks is implicated in pathogenesis, including neurodegenerative disease. In the mammalian endoplasmic reticulum (ER), more than 20 members of the protein disulfide isomerase family (PDIs) are believed to cooperate in the client folding pathway, but it remains unclear whether complex formation among PDIs via non-covalent interaction is involved in regulating their enzymatic and chaperone functions. Herein, we report novel functional hetero complexes between PDIs that promote oxidative folding and inhibit aggregation along client folding. The findings provide insight into the physiological significance of disulfide-catalyst and chaperone networks and clues for understanding pathogenesis associated with disruption of the networks.

**Abstract:**

P5 is one of protein disulfide isomerase family proteins (PDIs) involved in endoplasmic reticulum (ER) protein quality control that assists oxidative folding, inhibits protein aggregation, and regulates the unfolded protein response. P5 reportedly interacts with other PDIs via intermolecular disulfide bonds in cultured cells, but it remains unclear whether complex formation between P5 and other PDIs is involved in regulating enzymatic and chaperone functions. Herein, we established the far-western blot method to detect non-covalent interactions between P5 and other PDIs and found that PDI and ERp72 are partner proteins of P5. The enzymatic activity of P5-mediated oxidative folding is up-regulated by PDI, while the chaperone activity of P5 is stimulated by ERp72. These findings shed light on the mechanism by which the complex formations among PDIs drive to synergistically accelerate protein folding and prevents aggregation. This knowledge has implications for understanding misfolding-related pathology.

## 1. Introduction

Numerous amount of secretory and membrane proteins that are inserted into the mammalian endoplasmic reticulum (ER) must undergo disulfide bond formation-coupled protein folding, oxidative folding, to form unique functional three-dimensional structures [1,2,3]. Non-native disulfide-bonded misfolded proteins frequently lead to the formation of aggregates [4,5,6,7]. To reduce the risk of accumulation of aberrant proteins, almost all biological kingdoms have evolved both disulfide-catalyst and chaperone networks that assist proper folding [8,9,10]. Correspondingly, more than 20 members of the protein disulfide isomerase family (PDIs), e.g., PDI, ERp57, ERp72, ERp46, and P5, and other ER-resident chaperones constitute a protein homeostasis network that catalyzes oxidative folding and inhibits aggregation to ensure proper protein production in the mammalian ER [11,12,13,14,15]. Thus, mutation or unfavorable post-translational modification of PDIs is associated with misfolding diseases, such as amyotrophic lateral sclerosis, Alzheimer’s disease, Parkinson’s disease, and prion disease [16,17,18,19,20]. Although many researchers have vigorously investigated the disulfide-catalyst and molecular chaperone activities of PDI [21,22,23,24,25,26,27,28,29,30], the underlying mechanism of how other PDIs modulate the assistance of oxidative folding and the inhibition of client aggregation remains unclear [31].

Some substrates and enzymes that modulate PDI functions have been reported. Unfolded substrate-induced PDI homo-dimerization enhances the ability of disulfide-introduction into substrates [32]. Additionally, the α-subunit of prolyl 4-hydroxylase (P4HA)-bound PDI (also known as P4HB) is involved in procollagen maturation process [33,34]. Similarly, PDI complexed with the α-subunit of microsomal triglyceride transfer protein functions as a lipid transfer machine [12,35]. These examples demonstrate that the activities and functions of PDIs are regulated by forming homo- and hetero-complexes.

**Figure 1:**
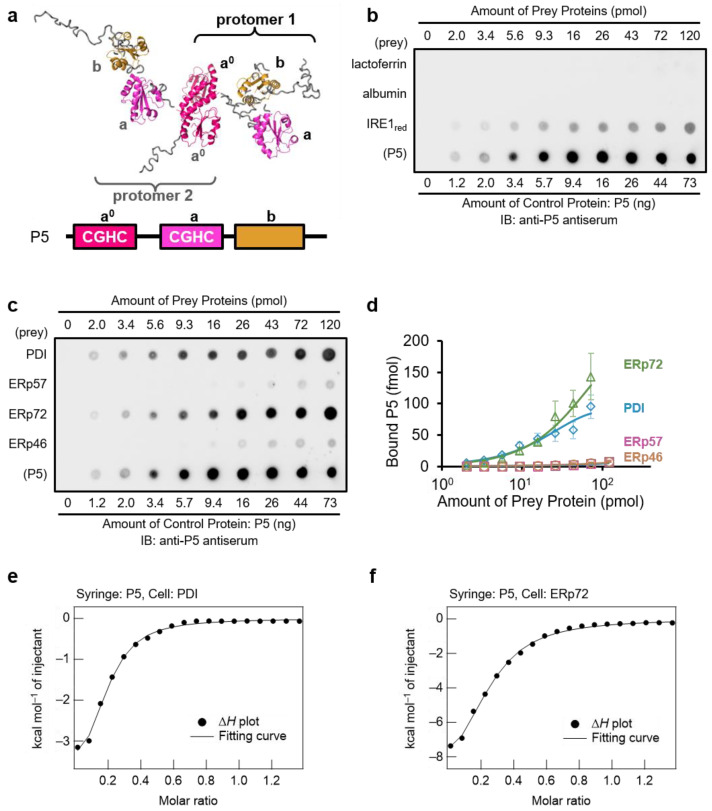
PDI and ERp72 are partner proteins of P5 that interact via non-covalent complex formation. (**a**) P5 structure [36]. P5 forms a dimer via the a^0^ domain (the first Trx-like domain). (**b**,**c**) Far-western dot blot analysis of non-covalent interactions between P5 and IRE1. Prey proteins, lactoferrin, albumin (negative control), IRE1 (positive control), and PDIs were dotted onto nitrocellulose membranes. As a control, bait protein (P5) was also dotted onto the bottom lane of the same membranes. Blocked membranes were incubated in P5 bait buffer, and bound P5 was detected using anti-P5 antisera. (**d**) Quantification of chemiluminescence signals in (**c**). The amount of P5 bound to PDIs on membranes was calculated from three independent experiments, compared with the signal intensities of control dots of P5 (bottom lane). Data are presented as means ± standard error of the mean (SEM). (**e**,**f**) Binding isotherms for titration of P5 against PDI (**e**) and ERp72 (**f**). Catalytically-inactive P5 (Cys55/58/190/193Ala) mutant (280 μM) in the syringe was titrated into 40 μM PDI or 40 μM ERp72 in the cell.

Like PDI, P5 (also known as ERp5 or PDIA6) is one of PDIs involved in ER protein quality control that assists oxidative folding [13,14,36,37,38], inhibits protein aggregation [36,37], and regulates the ER stress response [16,36,39,40,41]. P5 consists of three thioredoxin (Trx)-like domains (Figure 1a) [11,16] and dimerizes via a unique leucine-valine adhesive motif contained in the N-terminal Trx-like domain [36]. This motif is involved in the regulation of its chaperone activity and the regulation of intermolecular disulfide bonds in the ER stress sensor IRE1 [36]. In addition, P5 reportedly interacts with other PDIs (PDI, ERp57, ERp72, and ERp46) via intermolecular disulfide bonds in cultured cells [42], suggesting that P5 and partner proteins may work cooperatively to catalyze oxidative folding in the ER. Despite this progress, it remains unclear whether complex formation between P5 and these PDIs is involved in the regulation of enzymatic and chaperone functions.

In this study, we performed comprehensive protein-protein interaction analysis using the far-western dot blot method and identified PDI and ERp72 as partner proteins of P5 that interact via non-covalent interactions. We also found that P5 cooperates with PDI to synergistically accelerate oxidative folding, while P5 cooperates with ERp72 to enhance chaperone activity. These results provide new insights into the functional significance of heterogeneous non-covalent complexes between PDIs that control protein homeostasis in the mammalian ER.

## 2. Materials and Methods

### 2.1. Antibodies and Reagents

Rabbit anti-P5 antiserum was prepared using recombinant P5. Antibodies were from the following sources: anti-rabbit (Cat. No. 711-035-152; Jackson ImmunoResearch Laboratories, West Grove, PA, USA); bovine serum albumin (Cat. No. A7638; Sigma-Aldrich, St. Louis, MO, USA); bovine milk lactoferrin (Cat. No. 123-04124; Fujifilm Wako Pure Chemical, Osaka, Japan); bovine pancreas ribonuclease (RNase A; Cat. No. R5500; Sigma-Aldrich); Glyceraldehyde-3-phosphate Dehydrogenase from rabbit muscle (GAPDH; Cat. No. G2267; Sigma-Aldrich).

### 2.2. Plasmid Construction

The human P5 (Lys20–Leu440) and ERp57 (Ser25–Leu481) genes cloned into pET15b were a kind gift from Dr. Masakazu Kikuchi (Ritsumeikan University). The human PDI (Asp18–Leu508), ERp46 (Arg33–Leu432), and ERp72 (Glu25–Leu645) genes were cloned into pET15b, as previously described [14,43]. The human IRE1 (Ser24–Thr446) gene was cloned into pGEX6p-1, as previously described [36].

### 2.3. Recombinant Protein Expression and Purification

The 6× His-tagged PDIs and mutated P5 were overexpressed in *Escherichia coli* strain BL21 (DE3) and purified. Expression of recombinant proteins was induced by the addition of 0.5 mM isopropyl-β-D-thiogalactopyranoside and culturing at 20 °C overnight. Cells were disrupted by a Branson SFX250 Digital Sonifier homogenizer (Branson Ultrasonics, Danbury, CT, USA) in buffer containing 50 mM Tris-HCl (pH 8.0) and 0.3 M NaCl. After clarification of the cell lysate by centrifugation (20,000× *g* for 20 min), the supernatant was purified by Ni-NTA column chromatography (Qiagen, Hilden, Germany), followed by digestion with Thrombin Protease (Cat. No. 33842-44; Nacalai Tesque, Kyoto, Japan) and 10 mM dithiothreitol treatment and then further purification by RESOURCE Q 6 mL column chromatography (GE Healthcare Life Science, Uppsala, Sweden). Finally, the sample was purified by Superdex 200 increase 10/300 GL column chromatography (GE Healthcare Life Science) pre-equilibrated with buffer containing 50 mM HEPES (pH 7.2) and 150 mM NaCl.

C-terminal Glutathione S-transferase (GST) and 6× His-tagged IRE1α LD was overexpressed in *E. coli* strain BL21 (DE3) and purified. Expression of recombinant proteins was induced by addition of 0.5 mM isopropyl-β-D-thiogalactopyranoside and culturing at 15 °C overnight. Cells were disrupted by an NS1001L 2K homogenizer (Niro Soavi, Parma, Italy) in buffer containing 25 mM HEPES (pH 7.2), 25 mM L-arginine, 30 mM NaCl, and 10% (*w/v*) glycerol. After clarification of the cell lysate by centrifugation (18,000× *g* for 30 min), the supernatant was purified by Glutathione Sepharose 4B column chromatography (GE Healthcare Life Science), followed by digestion with PreScission Protease (GE Healthcare Life Science). The GST and 6× His-tag fusions were removed from digested IRE1α LD using Ni-NTA resin and further purification by RESOURCE Q 6 mL column chromatography (GE Healthcare Life Science). Finally, the sample was purified by Superdex 200 increase 10/300 GL column chromatography (GE Healthcare Life Science) pre-equilibrated with buffer containing 25 mM HEPES (pH 7.2), 25 mM L-arginine, and 150 mM NaCl.

### 2.4. Far-Western Dot Blotting

Far-western dot blot analyses were performed by optimizing the method reported in previous studies [44,45]. Reduced purified protein solutions (40 µM) were diluted to an appropriate concentration, and a 3 µL aliquot was dotted onto a ClearTrans nitrocellulose membrane (Cat. No. 037-25653; Fujifilm Wako Pure Chemical, Osaka, Japan) as prey protein. As a control experiment to estimate the amount of bound bait protein (P5), reduced bait protein (0.5 µM) was dotted onto the bottom lane of the same membrane in the amounts shown in Figure 1b,c. The membrane was blocked with 20 mM Tris-HCl (pH 7.6) containing 150 mM NaCl, 0.05% Tween 20, and 5% (*w/v*) skim milk (5% MTBST) at 4 °C for 1 h and then incubated in 0.2 μM of reduced P5 as bait protein in 20 mM Tris-HCl (pH 7.6) containing 150 mM NaCl and 0.05% Tween 20 (TBST) at 4 °C for 3 h. The membrane was washed three times with TBST and incubated with anti-P5 antiserum (1:10,000 in 1% MTBST). Next, the membrane was washed three times with TBST and incubated with horseradish peroxidase-conjugated anti-rabbit secondary antibody (1:10,000 in 1% MTBST). The membrane was washed four times with TBST, and signals were visualized using Chemi-Lumi One Super (Nacalai Tesque). Luminescence images were obtained by a LAS 4000 mini-instrument (Fuji Film), and relative signal intensities were quantified compared with signals of bait control protein (Figure 1c, P5) with ImageJ/Fiji [46], Microsoft Excel (Microsoft, Roselle, IL, USA), and GraphPad Prism 6.07 (GraphPad Software, Inc., La Jolla, CA, USA). The amount of P5 bound to prey proteins was calculated from the relative signal intensities according to the calibration curve obtained from the signals of bait control protein (P5).

### 2.5. Isothermal Titration Calorimetry

Isothermal titration calorimetry (ITC) measurements of PDIs complex formation were carried out with 40 μM PDIs dissolved in 50 mM HEPES (pH 7.2) using a MicroCal Auto-iTC200 instrument (Malvern Panalytical, Malvern, UK) with continuous stirring at 750 rpm. Next, 110 μL of 50 mM HEPES (pH 7.2) and 150 mM NaCl solution containing 280 μM of the P5 Cys55/58/190/193Ala mutant in the syringe were consecutively injected into the PDIs solution in the cell after a 60 s initial delay for thermal equilibration. The total number of titrations was 19 and the titration interval was 150 s. The initial delay and reference power were set to 60 s and 10 μcal·s^−1^, respectively. The first titration was conducted using 0.4 μL of solution, and the remaining titrations was 2 μL in the syringe. Changes in the heat flow were observed in real time at 10 μcal s^−1^ reference power and 30 °C. The dilution heat of P5 Cys55/58/190/193Ala mutant was also measured using the same method and the ITC parameters. The binding isotherms after the subtraction of dilution heat and the baseline correction were fitted to one set of site binding model incorporated in the MicroCal Origin 7.0 software. ([47]):

$$\begin{array}{l} Q\\ =\frac{n{\left[\mathrm{PDIs}\right]}_{t}\Delta HV_{0}}{2}[1+\frac{{\left[P5\;\mathrm{Cys}55/58/190/193\mathrm{Ala}\right]}_{t}}{n{\left[\mathrm{PDIs}\right]}_{t}}+\frac{K_{d}}{n{\left[\mathrm{PDIs}\right]}_{t}}\\ -\sqrt{{\left(1+\frac{{\left[P5\;\mathrm{Cys}55/58/190/193\mathrm{Ala}\right]}_{t}}{n{\left[\mathrm{PDIs}\right]}_{t}}+\frac{K_{d}}{n{\left[\mathrm{PDIs}\right]}_{t}}\right)}^{2}-\frac{4{\left[P5\;\mathrm{Cys}55/58/190/193\mathrm{Ala}\right]}_{t}}{n{\left[\mathrm{PDIs}\right]}_{t}}}] \end{array}$$

where *Q* is the total heat constant and *n* indicates the number of P5 Cys55/58/190/193Ala mutant molecules, which bind to one of the PDIs. ∆*H* is the change in enthalpy for the intermolecular interactions. *V*_0_ is an active cell volume, which considers the volume of the ITC cell and the volume increased by titration. The dissociation constant is denoted as *K*_d_. The total concentrations of P5 Cys55/58/190/193Ala mutant and PDIs are displayed as [P5 Cys55/58/190/193Ala]_t_ and [PDIs]_t_, respectively, at any given time (t).

### 2.6. Preparation of Reduced and Denatured RNase A

Reduced and denatured RNase A was prepared as previously described [44,48]. Briefly, RNase A was dissolved in buffer consisting of 50 mM Tris-HCl (pH 8.0), 6 M GdnHCl, and 40 mM DTT and incubated for 2 h at 25 °C. The incubated sample was dialyzed three times with a large excess of 10 mM HCl to remove the denaturing and reducing reagents.

### 2.7. Gel-Shift Assay of RNase A Oxidation

Gel-shift assays were performed by optimizing the method reported in previous studies [44,49]. Reduced and denatured RNase A (16 µM) was incubated with refolding buffer containing 50 mM HEPES (pH 7.2), 150 mM NaCl, 1 mM reduced glutathione (GSH), and 0.2 mM oxidized glutathione (GSSG), in the absence/presence of 1 µM PDIs or 0.5 µM P5 and other PDIs. At selected time points, the reaction was quenched, and free thiols were modified by addition of Laemmli 4 × sodium dodecyl sulfate (SDS)-sample buffer [32] containing 10 mM methoxy-polyethylene glycol maleimide MW 2000 (mPEG-mal 2k; Cat. No. ME-020MA; NOF America Corporation, White Plains, NY, USA). Modified samples were separated by nonreducing 14% SDS-polyacrylamide gel electrophoresis (PAGE) using a WIDE RANGE gel (Nacalai Tesque). Proteins were stained with Coomassie Brilliant Blue G250 (Nacalai Tesque) [50]. Band intensities were analyzed using ImageJ/Fiji [46] and Microsoft Excel. The relative band intensities of fully reduced form (**R**) and fully oxidized form (**N/4SS**) on SDS-PAGE gels were calculated compared with the band intensity of negative controls (**0**) and positive controls (**N**) in the same gels, respectively.

### 2.8. RNase A Reactivation Assay

Reactivation assays were performed by optimizing the method reported in previous studies [44,48,51]. Reduced and denatured RNase A (16 µM) was incubated with refolding buffer containing 50 mM HEPES (pH 7.2), 150 mM NaCl, 1 mM GSH, and 0.2 mM GSSG, in the absence/presence of 1 µM PDIs or 0.5 µM P5 and 0.5 µM other PDIs. At selected time points, 50 μL aliquots were taken from the refolding mixture and immediately diluted with 150 μL reaction buffer containing 50 mM HEPES (pH 7.2), 150 mM NaCl, and 0.8 mM cytidine 2′,3′-cyclic monophosphate (cCMP) monosodium salt (final concentration of cCMP = 0.6 mM; Cat. No. C9630; Sigma-Aldrich). The absorbance at 284 nm was measured with a V-730BIO spectrophotometer (JASCO Corp., Tokyo, Japan). The reaction rate of cCMP hydrolysis was calculated from the linear increase in the absorbance. The ratio of refolded RNase A was calculated from the relative rate compared with the reaction rate of native RNase A.

### 2.9. GAPDH Aggregation Assay

GAPDH was dissolved in buffer comprising 50 mM Tris-HCl (pH 8.0), 3 M GdnHCl, and 3 mM DTT and incubated overnight at 4 °C. Aggregation assays were performed by optimizing the method reported in previous studies [52,53,54]. Reduced and denatured GAPDH (125 µM) was diluted 125-fold (final concentration of GAPDH = 1 µM) in a chaperone assay buffer containing 50 mM HEPES (pH 7.2) and 150 mM NaCl and incubated at 20 °C in the absence/presence of 5 µM PDIs or 2.5 µM P5 and 2.5 µM other PDIs. Aggregation of GAPDH was monitored by 90° light scattering at 620 nm on a FP-8300 spectrofluorometer (JASCO Corp). Data were analyzed by Spectra Manager software (JASCO Corp) and Microsoft Excel. The initial rate of GAPDH aggregation was calculated from the increase in fluorescence intensity during the initial 60 s.

### 2.10. Statistical Analysis

Statistical analysis of the results of PDIs combinations and calculated arithmetic means of the results of P5 alone and PDIs alone was performed by Microsoft Excel using a two-tailed *t*-test, and *p* < 0.05 was considered significant.

## 3. Results

### 3.1. PDI and ERp72 Form Non-Covalent Complexes with P5

To investigate complex formation between P5 and other PDIs via non-covalent interactions, we used the far-western dot blot method [44,45,55,56] (Figure 1b,c). Briefly, we fixed known concentrations of reduced forms of PDIs as prey proteins onto a nitrocellulose membrane. After blocking and treatment with 0.2 μM reduced form of P5 as bait protein, the membrane was washed, and bait proteins (P5) bound to the prey proteins were detected using anti-P5 antiserum. There was no signal for P5 bound to the secretory proteins lactoferrin and albumin, but positive signals were observed for the ER stress sensor IRE1, which is regulated by P5 [36,39] (Figure 1b). As demonstrated in previous studies [21], this method could be applied for in vitro interactome analysis of P5 to identify partners binding via non-covalent interactions. We therefore examined whether non-covalent binding occurs between P5 and other PDIs using reduced PDIs for far-western dot blot analysis and found that PDIs bound to P5 with different affinities (Figure 1c). Quantification of chemiluminescence signals with defined amounts of prey proteins on membranes demonstrated that PDI and ERp72 were bound more tightly to P5 than ERp57 and ERp46 (Figure 1d). To quantitatively estimate the binding affinity of these intermolecular interactions in solution, isothermal titration calorimetry (ITC) experiments were performed (Figure 1e,f). P5 in the syringe was titrated into a solution containing ERp72 or PDI in the cell. The ITC thermograms of the interaction between P5 and ERp72/PDI indicated an exothermic reaction, suggesting that a negative enthalpy change contributed thermodynamically to the formation of complexes through intermolecular electrostatic and polar interactions (Figure 1e,f). Indeed, the ITC analyses revealed that the change in enthalpy (∆*H*) of P5 binding to PDI and ERp72 was −4.4 ± 0.2 and −10.7 ± 0.8 kcal mol^−1^, respectively. The change in entropy (∆*S*) was positive for PDI-P5 (*T*∆*S* = 3.4 ± 0.3 kcal mol^−1^); however, it was negative for PDI-ERp72 (*T*∆*S* = −3.3 ± 0.5 kcal mol^−1^). The *n* value indicating the binding stoichiometry was 0.10 ± 0.0 for both binding systems, which suggests that the same number of ERp72 and PDI molecules bind to P5 based on a one-site binding model. It was noteworthy that the affinity of P5 for PDI was similar to that for ERp72, based on the change in Gibbs free energy (∆*G*) of −7.5 ± 0.2 to −7.8 ± 0.1 kcal mol^−1^ and a dissociation constant (*K*_d_) ranging from 2.4 ± 0.3 to 4.1 ± 0.8 μM. Thus, we concluded that both ERp72 and PDI bind non-covalently to P5.

### 3.2. P5 and PDI Act in Concert to Synergistically Accelerate Oxidative Folding

**Figure 2:**
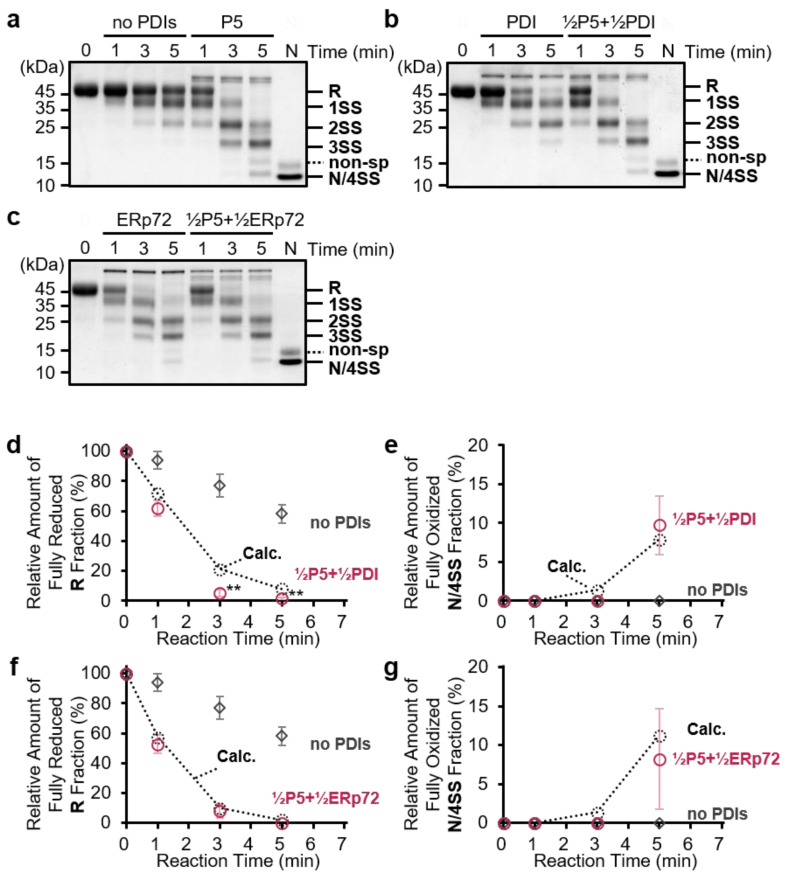
Disulfide bond formation in RNase A catalyzed by P5 in concert with PDI/ERp72. (**a**–**c**) RNase A oxidation assay in the presence of P5, PDI, ERp72, P5/PDI, and P5/ERp72. Reduced and denatured RNase A (16 µM) was incubated without PDIs or with 1 µM P5 (**a**); 1 µM PDI or 0.5 µM P5 and 0.5 µM PDI (**b**); or 1 µM ERp72 or 0.5 µM P5 and 0.5 µM ERp72 (**c**). In addition to reduced RNase A, an equal amount of native RNase A was loaded into lane N as a positive control. **R**, fully reduced form of RNase A; **1SS**, **2SS**, **3SS**, intermediates with one, two, or three disulfide bonds, respectively; **N/4SS**, mixture of the native form of RNase A and the fully oxidized form possessing four disulfide bonds; **non-sp**, non-specifically modified band. (**d**–**g**) Quantification of the relative band intensities of the fully reduced form (**R**: **d**,**f**) and fully oxidized form (**N/4SS**: **e**,**g**) following SDS-PAGE analysis (**a**–**c**). Error bars indicate the means ± SEM of three independent experiments. **½P5+½PDI**: results for 0.5 µM P5 and 0.5 µM PDI; **½P5+½ERp72,** results for 0.5 µM P5 and 0.5 µM ERp72; **no** **PDIs**, results without PDIs; **Calc.**, calculated arithmetic means of the results of P5 alone and PDIs alone (see Figure S1). ** *p* < 0.01, statistical significance between the means of **½P5+½PDIs** and **Calc.** using a two-tailed *t*-test.

To estimate the impact of PDI/ERp72 on P5 enzymatic function, we next investigated whether the combination of P5-PDI or P5-ERp72 affects the efficiency of disulfide bond introduction. Ribonuclease A (RNase A) containing four disulfide bonds was used as a model substrate. Reduced and denatured RNase A was incubated with 1 µM PDIs alone, and free thiol(s) in RNase A were modified with methoxy-polyethylene glycol maleimide MW 2000 (mPEG-mal 2k). The slower migration of RNase A modified by mPEG-mal 2k can be used to separate redox states by SDS-PAGE according to the number of modifications via free thiol(s) (Figure 2). In the presence of P5, reduced RNase A (**R**) disappeared at 3 min, and fully-oxidized RNase A (**N/4SS**) was observed at 5 min during an early folding step (Figure 2a). However, fully-oxidized RNase A (**N/4SS**) was hardly visible at 5 min in the presence of PDI (Figure 2b,c). Consistent with our previous results [14,36], P5 introduced disulfide bonds into clients more rapidly than PDI and ERp72.

To investigate whether the intermolecular interactions between PDIs are related to enzymatic efficiency, we performed a series of combination experiments. The catalytic efficiency was evaluated by comparing the theoretical values (calculated arithmetic means of the results of each 1 µM PDIs alone from Figure S1) with the experimental values of the combinations at half concentrations (0.5 µM P5 + 0.5 µM other PDIs). If intermolecular interactions affect the catalytic efficiency, there should be a significant difference between theoretical and experimental values. In the case of the P5-PDI combination, the band corresponding to reduced RNase A disappeared within 3 min, and oxidized RNase A was visible at 5 min (Figure 2b; **½P5+½PDI**). Quantitative analysis showed that, with this combination, the band intensity of reduced RNase A at both 3 min and 5 min was significantly lower than the theoretical value (Figure 2d; **½P5+½PDI** and **Calc.**), implying synergistic introduction of disulfide bonds by P5-PDI during the initial step. In the case of the P5-ERp72 combination, the band corresponding to reduced RNase A also disappeared within 3 min, but the effect appeared to be similar to that of P5 alone and ERp72 alone (Figure 2c). In contrast to the P5-PDI combination, quantitative analysis demonstrated that the P5-ERp72 combination had no impact on disulfide bond formation compared with the theoretical value (Figure 2g; **½P5+½ERp72** and **Calc.**).

**Figure 3:**
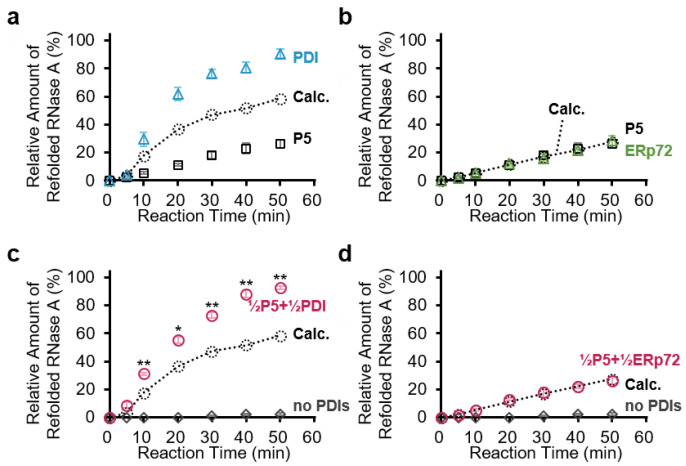
P5 acts in concert with PDI to synergistically accelerate oxidative folding of RNase A. (**a**–**d**) Reduced and denatured RNase A (16 µM) was incubated without PDIs (**no PDIs**), with 1.0 µM P5 (**a**,**b**), 1.0 µM PDI (**a**), 1.0 µM ERp72 (**b**), a mixture of 0.5 µM P5 and 0.5 µM PDI (**½P5+½PDI**) (**c**), or a mixture of 0.5 µM P5 and 0.5 µM ERp72 (**½P5+½ERp72**) (**d**), and cCMP hydrolysis by refolded RNase A was measured. The ratio of refolded RNase A was calculated from the relative rate compared with the reaction rate of native RNase A. Error bars indicate the means ± SEM of three independent experiments. **Calc.**, calculated arithmetic means of the relative rates of P5 alone and PDIs alone (dotted line and circle). *, *p* < 0.05; **, *p* < 0.01, statistical significance between the mean of **½P5+½PDI**, **½P5+½ERp72** and **Calc.** by two-tailed *t*-test.

To determine whether oxidized RNase A folds into a native conformation, we measured the hydrolysis activity of refolded RNase A using cytidine 2′,3′-cyclic monophosphate (cCMP) as a substrate. Compared with the theoretical value calculated using the results of P5 alone and PDI alone (Figure 3a), the ratio of refolded RNase A catalyzed by the combination of PDI-P5 was markedly higher than the theoretical value (Figure 3c; **½P5+½PDI** and **Calc.**), implying synergistic acceleration of oxidative folding by the P5-PDI combination. On the other hand, the P5-ERp72 combination had no impact on disulfide bond formation compared with the theoretical value (Figure 3b,d).

### 3.3. P5 Acts in Concert with ERp72 to Strongly Inhibit Client Aggregation

**Figure 4:**
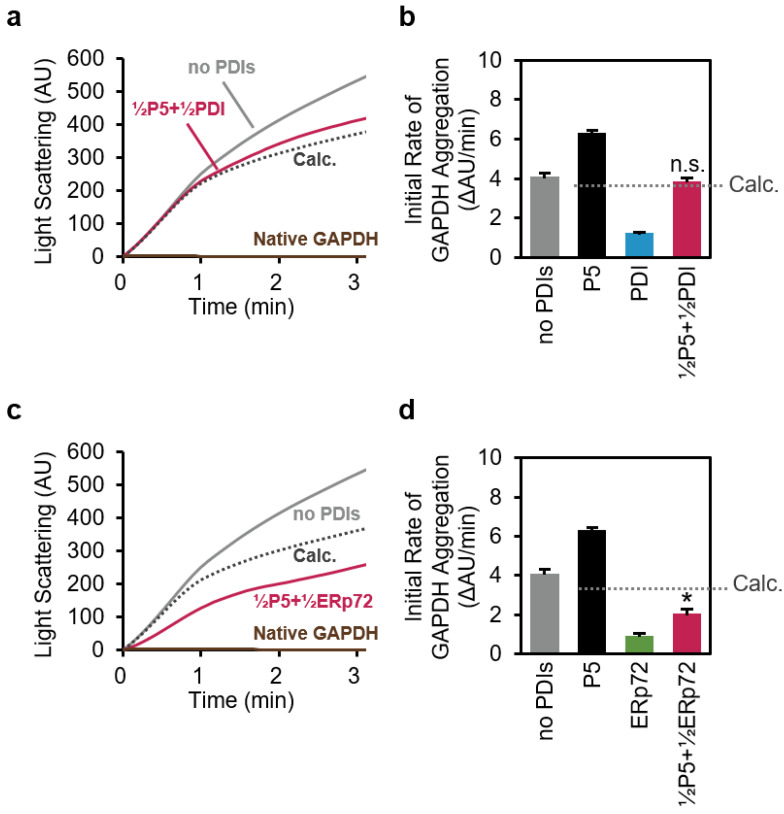
P5 acts in concert with ERp72 to strongly inhibit client aggregation. (**a**,**c**) Reduced and denatured GAPDH (1 µM) was incubated without PDIs (**no PDIs**), with 2.5 µM P5 and 2.5 µM PDI (**½P5+½PDI**), or with 2.5 µM P5 and 2.5 µM ERp72 (**½P5+½ERp72**), and GAPDH aggregation was evaluated by measuring 90° light scattering at 620 nm over time. As a control, folded and non-reduced GAPDH (**Native GAPDH**) was also incubated and evaluated. (**b**,**d**) Comparison of aggregation rates in (**a**,**c**). The initial rate of GAPDH aggregation was calculated from the increase in fluorescence intensity during the initial 60 sec. Error bars indicate the means ± SEM of three independent experiments. AU, arbitrary units; **Calc.**, calculated arithmetic means of the initial rates of P5 and PDIs (see Figure S2). *, *p* < 0.05, statistical significance between the mean of **½P5+½PDI** or **½P5+½ERp72** and **Calc.** by a two-tailed *t*-test; n.s., no statistical significance.

Next, we examined chaperone activity using reduced/denatured GAPDH aggregation assays (Figure S2 and Figure 4). As a control, non-reduced and folded GAPDH (**Native GAPDH**) did not exhibit light scattering, whereas reduced/denatured GAPDH without PDIs (**noPDIs**) showed a significant increase in light scattering intensity (Figure 4a). The result indicated that P5 induces reduced/denatured GAPDH to aggregate (Figure S2), while PDI or ERp72 inhibits aggregation of GAPDH (Figure S2a,b and Figure 4b,d). We subsequently calculated theoretical profiles using arithmetic means (**Calc.**). The light scattering profile of P5 in concert with PDI (**½P5+½PDI**) was almost the same as the theoretical profile, suggesting that, despite the complex formation, PDI did not affect the property of P5 to induce GAPDH aggregation.

Importantly, the GAPDH aggregation was strongly inhibited by P5 in concert with ERp72 (**½P5+½ERp72**) compared with the arithmetic mean (Figure 4c). Of note, the initial aggregation rate was also significantly decreased compared with the arithmetic mean calculated from the initial rates of P5 and ERp72 (Figure 4d). This result suggests that P5-induced GAPDH aggregation is suppressed by ERp72 and that the P5 chaperone activity is increased by forming a complex with ERp72.

## 4. Discussion

**Figure 5:**
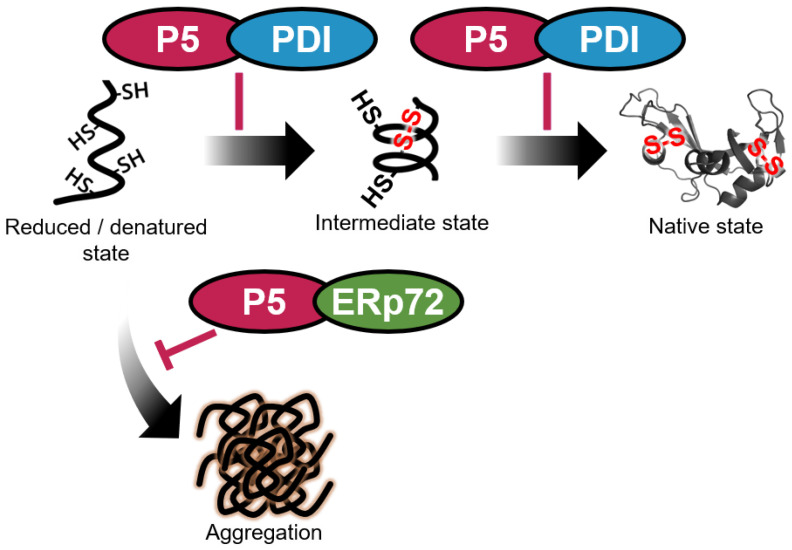
Schematic representation of the efficient productive folding system resulting from non-covalent interaction between P5, PDI, and ERp72. The combination of P5 and PDI enhances the efficiency of disulfide bond formation and isomerization during the oxidative folding reaction. Meanwhile, the combination of P5 and ERp72 efficiently inhibits aggregation of reduced and denatured substrates in the initial folding step. Straight and curved arrows indicate the progression of disulfide bond formation and isomerization during the oxidative folding reaction and the progression of misfolding and aggregation of unfolded substrates, respectively.

In this study, we showed that P5 non-covalently interacts with PDI (Figure 1) to enhance the efficiency of disulfide bond formation and isomerization (Figure 3 and Figure 4). Previous studies demonstrated that P5 binds to PDIs via covalent interactions in cultured cells [42] and that the plant P5 homolog promotes oxidative folding by transfer of disulfide bonds to the plant ERp57 homolog [44]. These findings suggest that disulfide-bonded complexes between PDIs promote oxidative client folding, but knowledge on non-covalent complexes between PDIs remains limited. Some studies on non-covalent PDI complexes have been reported; the oxidized form of PDI forms non-covalent homodimers in an unfolded substrate-dependent manner to promote oxidative folding [32], and PDI also binds to P4HA and MTP to facilitate other functions of the partners by forming hetero-complexes [12,33,34,35]. Our current results demonstrate that hetero-complex formation between P5 and PDI via non-covalent interaction plays a key role in stimulating enzymatic activity (Figure 5).

Our previous works demonstrated that, while disulfide bonds are rapidly and promiscuously introduced by P5/ERp46 during the early stage of oxidative protein folding, PDI efficiently proofreads non-native disulfide bonds during the later stage [10,11]. As a result of the synergistic effects of these enzymes, oxidative protein folding can be greatly accelerated. The present work provides another mechanistic implication: P5 and PDI can work cooperatively to accelerate oxidative protein folding by forming a non-covalent complex (Figure 1). Further biophysical and cell biological experiments are awaited to elucidate molecular and physiological details of the functional interplays between P5 and PDI.

Regarding chaperone function, the efficiency was increased by P5-ERp72 combination (Figure 4 and Figure 5). During the catalysis of initial oxidative folding process, P5 appears to gain easy access to a random coiled aggregation-prone conformation [14,36]. Nevertheless, only P5 displayed lower chaperone activity against reduced/denatured client (Figure S2) but relatively higher chaperone activity against folded client [36]. When interpreted in terms of client folding, the P5 chaperone function seems to be enhanced by forming a complex with ERp72 during the initial folding step. The efficiency of chaperones can be enhanced when they interact with each other, not only in the ER [57,58], but also in other cellular compartments [59,60,61,62,63,64].

## 5. Conclusions

In conclusion, we identified P5-PDI and P5-ERp72 as functional hetero-complexes between PDIs that accelerate client folding. We propose a complex formation-driven modulation mechanism of enzymatic and chaperone activities that has implications for anti-aggregation, productive folding, and misfolding-related pathology.

## Data Availability

The authors declare that all data supporting the findings of this study are available within the paper. All other information is available from the corresponding authors upon reasonable request.

## Supplementary Materials

The following are available online at https://www.mdpi.com/article/10.3390/biology10111112/s1: Figure S1, Disulfide bond formation of RNase A catalyzed by P5, PDI, or ERp72, related to Figure 2; Figure S2, Aggregation of GAPDH in the presence of P5, PDI, or ERp72, related to Figure 4.
