# Supplementary material for: Functional Interplay between P5 and PDI/ERp72 to Drive Protein Folding
Source: Biology (Basel). 2021 Oct 28;10(11):1112. doi: 10.3390/biology10111112 (PMC8615271; doi:10.3390/biology10111112)
Supplement: Supplementary file 1 [file biology-10-01112-s001.zip › biology-1420891-supplementary.pdf]

biology

*Supplementary Materials*

# Functional Interplay between P5 and PDI/ERp72 to Drive Protein Folding

**Motonori Matsusaki, Rina Okada, Yuya Tanikawa, Shingo Kanemura, Dai Ito, Yuxi Lin, Mai Watabe, Hiroshi Yamaguchi, Tomohide Saio, Young-Ho Lee, Kenji Inaba, and Masaki Okumura***

* Correspondence: okmasaki@tohoku.ac.jp; Tel.: +81-22-795-5764

This PDF file includes:

Figure S1, Disulfide bond formation of RNase A catalyzed by P5, PDI, or ERp72, related to Figure 2; Figure S2, Aggregation of GAPDH in the presence of P5, PDI, or ERp72, related to Figure 4.

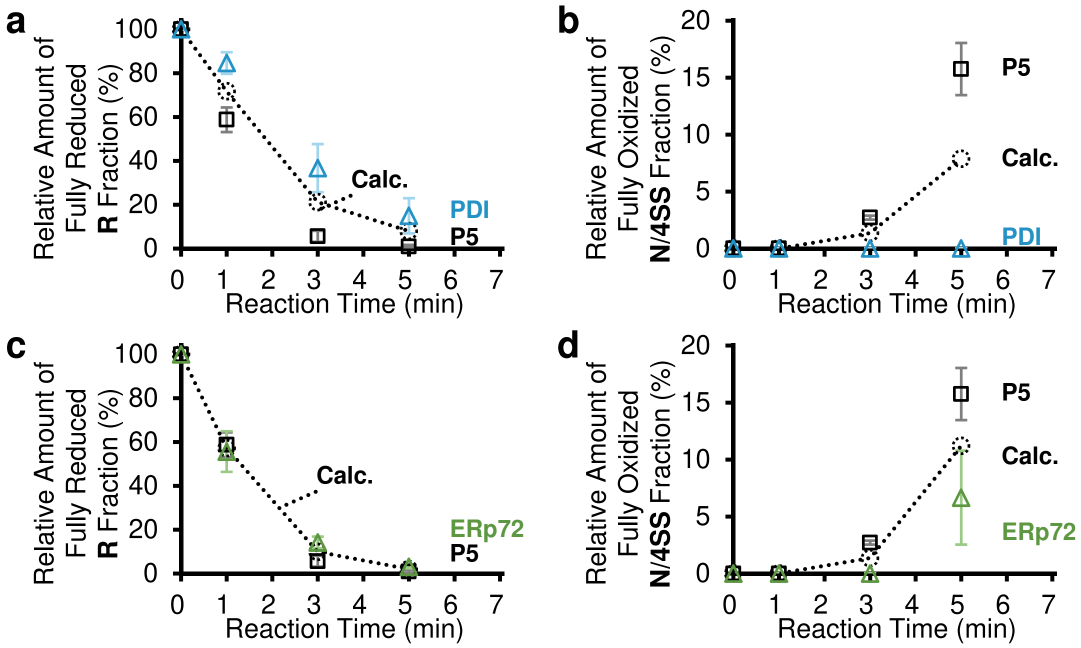


**Figure S1.** Disulfide bond formation of RNase A catalyzed by P5, PDI, or ERp72, related to Figure 2. (**a**–**d**) Quantification of the relative band intensities of reduced form (**a**,**c**) and oxidized form (**b**,**d**) in Figure 2. Error bars indicate the means ± SEM of three independent experiments. **P5**, results of 1 μM P5 alone; **PDI**, results of 1 μM PDI alone; **ERp72**, results of 1 μM ERp72 alone; **Calc.**, calculated arithmetic means of the results of P5 alone and PDIs alone (dotted line and circle).

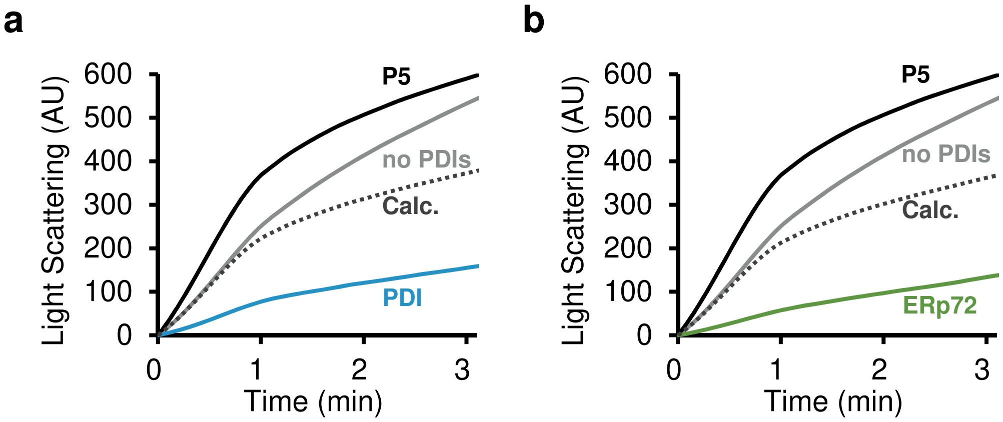


**Figure S2.** Aggregation of GAPDH in the presence of P5, PDI, or ERp72, related to Figure 4. (**a**,**b**) Reduced and denatured GAPDH (1 µM) was incubated without PDIs (**no PDIs**), with 5 µM P5 alone (**P5**), with 5 µM PDI alone (**PDI**), or with 5 µM ERp72 alone (**ERp72**). GAPDH aggregation was evaluated by measuring 90˚ light scattering at 620 nm over time. AU, arbitrary units; **Calc.**, calculated arithmetic means of the fluorescence intensities of P5 alone and PDIs alone (dotted line).
